# A Preliminary Study of Frequency and Clinical Relevance of Cytotoxic Peripheral CD4 and CD8 T Cells in Patients with Anti-MDA5 Positive Dermatomyositis

**DOI:** 10.2478/rir-2022-0022

**Published:** 2022-10-20

**Authors:** Fengyun Jia, Shan Jiang, Jiamin Zhang, Qiong Fu, Xiaoming Zhang, Yan Ye

**Affiliations:** 1 The Center for Microbes, Development and Health, Key Laboratory of Molecular Virology and Immunology, Institut Pasteur of Shanghai/University of Chinese Academy of Sciences, Chinese Academy of Sciences, Shanghai 200031, China; 2 Blood Group Reference Laboratory, Shanghai Blood Center, Shanghai 200051, China; 3 Department of Rheumatology, Renji Hospital, Shanghai Jiaotong University School of Medicine, Shanghai 200001, China

**Keywords:** anti-melanoma differentiation-associated gene 5-positive dermatomyositis, cytotoxic T cells, granzyme, prognosis

## Abstract

**Objectives:**

Anti-melanoma differentiation-associated gene 5-positive dermatomyositis (MDA5^+^DM) is an autoimmune disease frequently accompanied by rapidly progressive interstitial lung disease (RP-ILD) with high mortality. T cells are implicated in the pathogenesis of MDA5^+^DM and this study aims to measure the frequency and clinical relevance of cytotoxic CD4 and CD8 T cells in this disease.

**Methods:**

T cells expressing Perforin, Granzyme B (GZMB) and Granzyme K (GZMK) were analyzed by flow cytometry from peripheral blood of 19 patients with active MDA5^+^DM and 19 age- and sex-matched healthy donors (HDs). The frequency of CD4 and CD8 T cells and the cytotoxic subsets were compared between patients with MDA5^+^DM and HDs. Correlations within T cell subsets and between T cell subsets and clinical parameters of lactate dehydrogenase (LDH), ferritin, neutrophil-to-lymphocyte ratio (NLR), and Myositis Intention-to-Treat Index (MITAX) were evaluated.

**Results:**

Compared with HDs, patients with active MDA5^+^DM significantly had increased frequency of CD4 T cells, and reduced frequency of GZMK^+^GZMB^−^ CD8 T cells. Furthermore, the frequency of GZMK^+^GZMB^−^ CD8 T cells positively correlated with serum ferritin levels in active MDA5^+^DM patients. Notably, the patients in the Dead group of MDA5^+^DM had a significant higher frequency of GZMK^+^GZMB^−^ CD4 and CD8 T cells.

**Conclusion:**

Substantial changes of cytotoxic T cell subsets are observed in active MDA5^+^DM patients. In addition, a high frequency of GZMK^+^GZMB^−^ CD4 and CD8 T cells is associated with unfavorable prognosis in MDA5^+^DM. More studies are warranted to further explore the roles of cytotoxic T cells in MDA5^+^DM.

## Introduction

Anti-melanoma differentiation-associated gene 5-positive dermatomyositis (MDA5^+^DM) is a rare but distinct subtype of dermatomyositis (DM), which is typically characterized by anti-MDA5 autoantibody, DM rash, multiple arthralgia, and interstitial lung disease (ILD), while the clinical signs of myositis are usually absent.^[1,2]^ Due to the high incidence of rapidly progressive interstitial lung disease (RP-ILD), MDA5^+^DM is usually associated with poor prognosis and high mortality, both in East Asia and Western countries.^[3,4,5,6,7]^

The etiology and pathogenesis of MDA5^+^DM have not been elucidated so far. However, several lines of evidence indicate that T cells may play a critical role in the immunopathogenesis. First, targeting T cells by using calcineurin inhibitors (CNIs) such as cyclosporine (CsA) A and tacrolimus (TAC) has shown promising efficacy in treating MDA5^+^DM.^[1,2,8]^ Second, a population of CD4^+^CXCR4^+^ T cells were increased in the blood and bronchoalveolar lavage fluid of MDA5^+^DM patients, and these cells could exert a promoting role on pulmonary fibroblast proliferation.^[9]^ Finally, we recently reported an overall activation of peripheral T cell compartment, particularly the CD8 T cell subsets in MDA5^+^DM patients.^[10]^

A major function of CD8 T cells is to kill target cells via their cytotoxic molecules such as Perforin and Granzymes.^[11]^ In this study, we performed a comprehensive analysis of the expression of main cytotoxic molecules in CD4 and CD8 T cells from active MDA5^+^DM patients and healthy donors (HDs), with the aim of identifying disease-associated cytotoxic T cell pattern and its potential clinical relevance in MDA5^+^DM.

## Materials and Methods

### Study Subjects

Peripheral blood samples were collected from 19 MDA5^+^DM-ILD patients who were admitted to the Department of Rheumatology in Renji Hospital from September 2021 to March 2022, and 19 age- and sex-matched HDs from the Renji Hospital Biobank. All the patients fulfilled the 2017 EULAR/ACR Classification Criteria for Idiopathic Inflammatory Myopathies. Detailed information about the patients is listed in Table 1.

**Table 1:** Clinical features of HDs and patients with MDA5^+^DM in the study.

	**HDs (*n* = 19)**	**MDA5^+^DM (*n* = 19)**		***P* value^#^**

		**Alive (*n* = 14)**	**Dead (*n* = 5)**	
Female (%)	12 (63%)	10 (71%)	5 (100%)	0.5304 [0.2707^a^]
Age (years, mean ± SD)	50 ± 16	50 ± 13	53 ± 5	0.5844 [0.8988^a^]
Anti-MDA5 (%)	/	14 (100%)	5 (100%)	>0.9999
Anti-Ro-52 (%)	/	2 (14%)	1 (20%)	>0.9999
Ferritin (ng/ml, mean ± SD)	/	1046 ± 875	1483 ± 1612	0.9644
LDH (U/L, mean ± SD)	/	299 ± 108	432 ± 112	0.0339
NLR (mean ± SD)	/	13 ± 15	60 ± 82	0.1299
MITAX		0.29 ± 0.12	0.46 ± 0.03	0.0079
**Treatments**				
Dose of GC (mg)		143.04 ± 276.36	112 ± 43.82	0.7059
GC+TAC, *n* (%)		9 (64%)	4 (80%)	>0.9999
GC+CsA, *n* (%)		2 (14%)	1 (20%)	>0.9999
GC+CTX, *n* (%)		1 (7%)	0 (0%)	>0.9999
GC+CsA+CTX, *n* (%)		1 (7%)	0 (0%)	>0.9999
GC+TOFA, *n* (%)		1 (7%)	0 (0%)	>0.9999

CTX, cyclophosphamide; CsA, cyclosporine; DM, dermatomyositis; GC, glucocorticoid; HDs, healthy donors; LDH, lactate dehydrogenase; MDA5, melanoma differentiation-associated gene 5; MITAX, myositis intention to treat activity index; NLR, neutrophil-to-Lymphocyte ratio; SD, standard deviation; TAC, tacrolimus; TOFA, tofacitinib.

a Statistical analysis is performed between the HD and MDA5^+^DM patients. Student's *t* test is used to compare the two groups.

# Statistical analysis is performed between the Alive and Dead groups of MDA5^+^DM patients if not indicated.

### Clinical Parameters

Serum, lactate dehydrogenase (LDH), ferritin, anti-MDA5, and anti-Ro-52, as well as neutrophil-to-lymphocyte ratio (NLR), were obtained from the clinical laboratory. Anti-MDA5 and anti-Ro-52 autoantibodies were detected by immunoblot testing following the manufacturer's instructions (Euroimmun, Lubeck, Germany). The gray-scale value of the antibody band was scanned to obtain semi-quantitative results, and gray-scale values of 0–10 were defined in the following way: 11–25 as +, 26–50 as ++, and >50 as +++. Myositis Intention-to-Treat Index (MITAX)^[12]^ is a disease activity score described by the International Myositis Assessment & Clinical Studies Group with 7 domains (cutaneous, muscle, constitutional, skeletal, gastrointestinal, pulmonary, and cardiovascular). The MITAX score was the sum score divided by the total score of 63 points, and the details of clinical information required for the calculation were obtained from the medical records. We defined a MITAX score of 0.14 (9/63) or higher as disease “active” and all 19 MDA5^+^DM patients recruited in this study were classified as “active” patients (Table 1).

### Flow Cytometry

Peripheral blood mononuclear cells (PBMCs) were isolated by density gradient centrifugation on Lymphoprep (Axis-shield). The following antibodies were used in flow cytometry: BUV395 anti-CD3 (clone SK7, BD, San Diego, CA), Brilliant Violet 570 anti-CD4 (clone RPA-T4, Biolegend, San Diego, CA), Alexa Fluor 700 anti-CD8a (clone HIT8a, Biolegend, San Diego, CA), PE anti-Granzyme B (clone GB11, BD, San Diego, CA), Alexa Fluor 647 anti-Granzyme K (clone G3H69, BD, San Diego, CA), and FITC anti-Perforin (clone B-D48, Biolegend, San Diego, CA).

PBMCs were first stained with Live/Dead dye FVS780 (BD, San Diego, CA) in PBS at room temperature for 30 min. Then, cells were stained with surface markers CD3, CD4, and CD8a in FACS buffer (1× PBS containing 1%FBS, 2.5 mM EDTA, and 0.1% NaN_3_) at room temperature for 15 min, followed by washing twice with FACS buffer. The cells were then fixed and subject to permeabilization with FOXP3 Fix/Perm buffer and FOXP3 perm buffer (eBioscience, San Diego, CA) at 4°C for 60 min. After this, they were washed twice with Foxp3 Perm buffer (eBioscience, San Diego, CA), and the intercellular Granzyme B, Granzyme K, and Perforin were stained with corresponding fluorochrome-conjugated antibodies at 4°C for 30 min in Foxp3 Perm buffer. Finally, the cells were washed twice with Foxp3 Perm buffer, filtered, and resuspended in FACS buffer before acquisition. Data were collected using a flow cytometry analyzer (LSRFortessa, BD, San Diego, CA) and analyzed using FlowJo Software (v.10.4, TreeStar, Ashland, OR).

### Statistical Analysis

Statistical analysis was performed using Prism 8.0.2 (GraphPad, San Diego, CA). Student's *t* test or Mann–Whitney test was used to compare the two groups as needed. Similarly, Spearman or Pearson's correlation coefficient was used to evaluate the correlation between the two groups as appropriate. A *P* value <0.05 was considered statistically significant.

## Results

### Characteristics of the Study Population

A total of 19 patients diagnosed with MDA5^+^DM and 19 healthy controls (HDs) were included in the study. All of these MDA5^+^DM patients were combined with ILD, and treated with 1–2 mg/kg methylprednisolone and CNIs. Five of them died within 6 months because of RP-ILD. There was no significant difference in age, serum ferritin levels, and NLR between the Alive and Dead groups (Table 1). However, the serum levels of LDH in the Dead group were significantly higher than those in the Alive group (Dead *vs.* Alive: 423 ± 112 U/L *vs.* 299 ± 10 U/L, *P* = 0.0339). Similarly, the MITAX scores were also significantly increased in the Dead group (Dead *vs.* Alive: 0.46 ± 0.03 *vs.* 0.29 ± 0.12, *P* = 0.0079) (Table 1). These results indicate that the Dead group shows a more severe disease status compared to the Alive group in MDA5^+^DM patients.

### Altered Frequency of Cytotoxic CD4 and CD8 T Cell Subsets in MDA5^+^DM Patients

**Figure 1:**
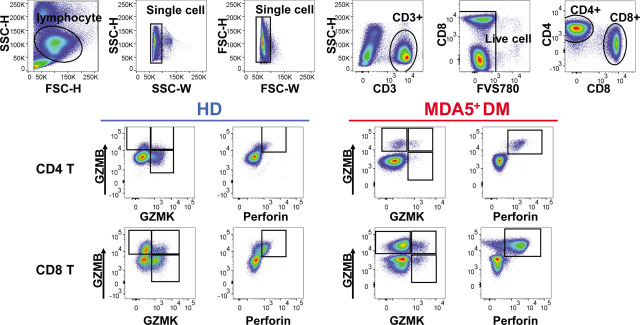
Gating strategy for flow cytometry analysis of peripheral cytotoxic CD4 and CD8 T cell subsets from HDs and MDA5^+^DM patients. DM, dermatomyositis; HDs, healthy donors; MDA5, melanoma differentiation-associated gene 5.

**Figure 2:**
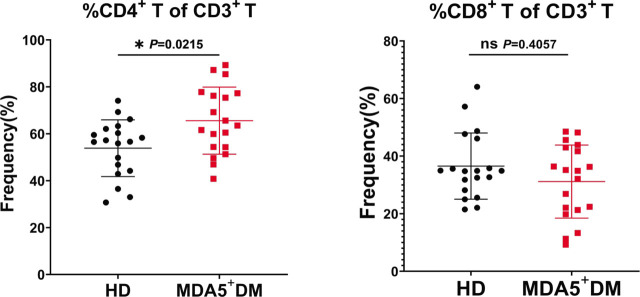
Comparison of the frequency of CD4 and CD8 T cells between HD and MDA5^+^DM groups. Statistical difference between HD and MDA5^+^DM groups was analyzed using Mann–Whitney test. ^*^P < 0.05. DM, dermatomyositis; HD, healthy donor; MDA5, melanoma differentiation-associated gene 5; ns: not significant.

A decrease of T cell counts^[13,14]^ and a strong activation of CD8 T cells^[10]^ have been reported in MDA5^+^DM patients. However, to our knowledge, the expression of cytotoxic molecules in T cells has not been explored in these patients. To this end, we developed a flow cytometric panel to measure key cytotoxic molecules Perforin, Granzyme K (GZMK), and Granzyme B (GZMB) in peripheral CD4 and CD8 T cells (Figure 1). We first observed that the frequency of CD4 T cells was significantly increased in MDA5^+^DM patients compared with that in HDs (MDA5^+^DM *vs.* HDs: mean±SD [same for the following], 65.59 ± 14.31% *vs.* 53.85 ± 12.09%, *P* = 0.0215) (Figure 2). The frequency of CD8 T cells showed a reverse tendency, but did not achieve significance (Figure 2). These results are largely consistent with the early reports indicating a decrease of CD8 T cells in MDA5^+^DM patients.^[13,14]^

**Figure 3:**
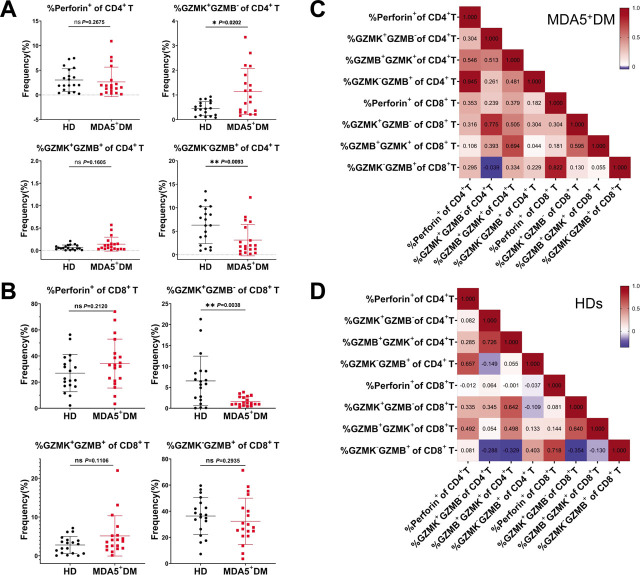
Comparison of the frequency of cytotoxic CD4 and CD8 T cell subsets between HD and MDA5^+^DM groups. The frequency of Perforin^+^, GZMK^+^GZMB^−^, GZMK^+^GZMB^+^, and GZMK^−^GZMB^+^ CD4 T cells (A) or CD8 T cells (B) was compared between HD (n = 19) and MDA5^+^DM (n = 19) groups. Statistical difference between the two groups was analyzed using Mann–Whitney test. (C and D) Heatmaps showing the correlations among cytotoxic CD4 and CD8 T cell subsets from MDA5^+^DM patients (C) and HDs (D). The numbers inside the heatmaps denote correlation coefficients. Spearman correlation analysis was applied. ^*^P < 0.05; ^**^P < 0.01. DM, dermatomyositis; GZMB, Granzyme B; GZMK, Granzyme K; HDs, healthy donors; MDA5, melanoma differentiation-associated gene 5; ns: not significant.

Next we compared the frequency of Perforin^+^, GZMK^+^GZMB^−^, GZMK^+^GZMB^+^, and GZMK^−^GZMB^+^ CD4 T cells or CD8 T cells between HD and MDA5^+^DM groups. We found that compared to HDs, the frequency of GZMK^+^GZMB^−^ CD4 T cells was significantly increased in MDA5^+^DM patients (HDs *vs.* MDA5^+^DM: 0.45 ± 0.28% *vs.* 1.34 ± 0.94%, *P* = 0.0202), and the frequency of GZMK^−^GZMB^+^ CD4 T cells was significantly decreased in MDA5^+^DM patients (HDs *vs.* MDA5^+^DM: 6.30 ± 3.93% *vs.* 3.12 ± 3.35%, *P* = 0.0093) (Figure 3A). Other CD4 T cell subsets showed no statistical difference between the two groups (Figure 3A). More differences were detected in CD8 T cells. In contrast to CD4 T cells, the frequency of GZMK^+^GZMB^−^ CD8 T cells was significantly lower in MDA5^+^DM patients than that in HDs (HDs *vs.* MDA5^+^DM: 6.53 ± 5.92% *vs.* 1.63 ± 1.13%, *P* = 0.0038) (Figure 3B). We also performed the correlation analyses among cytotoxic T cell subsets in MDA5^+^DM patients and HDs, and in general, we saw high correlations between cytotoxic CD4 and CD8 T cell subsets for both groups (Figures 3C and 3D). Collectively, the above results provide a comprehensive picture of cytotoxic molecule expression in CD4 and CD8 T cells from active MDA5^+^DM patients with reference to the data from HDs.

### Association of Cytotoxic T Cell Subsets with Clinical Features in Active MDA5^+^DM Patients

**Figure 4:**
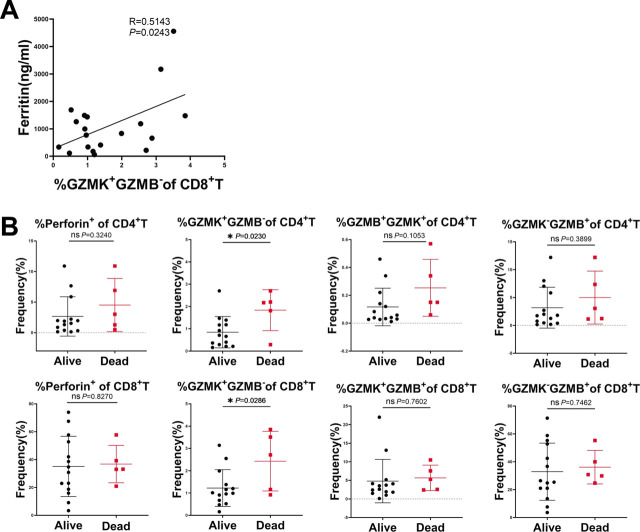
Association of cytotoxic CD4 and CD8 T cell subsets with clinical parameters in MDA5^+^DM patients. (A) Correlation analysis between serum ferritin levels and the frequency of GZMK^+^DM patients (n = 19). Pearson correlation analysis was applied. (B) Comparison of the frequency of cytotoxic CD4 and CD8 T cells between the Alive (n = 14) and Dead (n = 5) groups in MDA5^+^DM patients (n = 19). Statistical difference between the two groups was analyzed using Student's t test. ^*^P < 0.05. DM, dermatomyositis; GZMB, Granzyme B; GZMK, Granzyme K; MDA5, melanoma differentiation-associated gene 5; ns: not significant.

Finally, we evaluated the potential relationship between the frequency of cytotoxic T cell subsets and clinical parameters in MDA5^+^DM patients, including LDH, ferritin, NLR, MITAX, and prognosis. The data showed that the frequency of GZMK^+^GZMB^−^ CD8 T cells had a significantly positive correlation with serum ferritin levels in MDA5^+^DM patients (*R* = 0.5143, *P* = 0.0243) (Figure 4A). Notably, when the MDA5^+^DM patients were divided into Alive and Dead groups, the frequency of both GZMK^+^GZMB^−^ CD4 and GZMK+GZMB^−^CD8 T cells was significantly higher in the Dead group than in the Alive group (Dead *vs.* Alive: 1.8 ± 0.7% *vs.* 0.8 ± 0.7%; 2.4 ± 1.3% *vs.* 1.2 ± 0.85%, respectively) (Figure 4B). Altogether, our results suggest that both GZMK^+^GZMB^−^ CD4 and GZMK^+^GZMB^−^ CD8 T cells may have prognostic roles in active MDA5^+^DM patients.

## Discussion

MDA5^+^DM is a unique subtype of DM associated with RP-ILD and high mortality.^[1,2]^ In addition, the etiology and pathogenesis remain elusive and the treatment is largely empirical.^[7,8,15]^ Dysregulation of the T cell compartment is implicated in this disease,^[9,10,13,14]^ but direct evidence is largely lacking. In the current study, by profiling the cytotoxic CD4 and CD8 T cell subsets, we have provided an updated picture of disease-associated cytotoxic T cell pattern and the relationship with clinical parameters in active MDA5^+^DM patients.

The first finding of our study was that compared to the HDs, the frequency of CD4 T cells was increased while the frequency of CD8 frequency was decreased, and even the latter has not achieved statistical significance. This phenomenon is somewhat consistent with the early reports that CD8 T cells are decreased more significantly than CD4 T cells in patients with severe MDA5^+^DM.^[13,14]^ The reason for a more severe decrease of CD8 T cells is not clear, but given that CD8 T cells are highly activated in activated MDA5^+^DM patients,^[10]^ it is plausible that activated CD8 T cells could be recruited to affected tissues.

A close look at cytotoxic T cell subsets reveals more interesting findings. Among cytotoxic CD4 T cell subsets, the frequency of GZMK^+^GZMB^−^ CD4 T cells was increased in active MDA5^+^DM patients compared to HDs. By contrast, the frequency of GZMK^+^GZMB^−^ CD8 T cells was decreased in active MDA5^+^DM. The reason for the differential changes of GZMK^+^GZMB^−^ CD4 versus CD8 T cells is currently unknown.

Association analysis between cytotoxic T cell subsets and clinical parameters has uncovered a correlation between the frequency of GZMK^+^GZMB^−^ CD8 T cells and serum ferritin levels in active MDA5^+^DM patients. Furthermore, both the frequency of GZMK^+^GZMB^−^ CD4 and CD8 T cells was significantly increased in the Dead group compared to the Alive group of MDA5^+^DM patients. It has been reported that GZMK can induce signaling pathways associated with the onset of inflammation.^[16]^ Of note, increased frequency of GZMK+ CD8 T cells has been associated with inflammatory status across several physiological and pathological conditions, predominantly in the peripheral blood of aging people,^[17]^ in the synovial tissue of rheumatoid arthritis (RA) patients,^[18]^ and in the kidney of lupus nephritis patients.^[19]^ Clearly, more studies are needed to further explore the role of GZMK^+^ T cells in MDA5^+^DM patients.

### Limitations

We are aware that our current study has several limitations. First, the number of samples was relatively small and we only collected the blood samples at baseline in active MDA5^+^DM patients. Second, we did not include a cohort of “stable” MDA5^+^DM patients at remission. Third, we only detected the phenotypes of cytotoxic T cells without functional assays. We plan to address these limitations in our future exploration by recruiting a prospective cohort with follow-up samples.

## Conclusion

Cytotoxic T cell subsets are modified in active MDA5^+^DM patients. Furthermore, a high frequency of GZMK^+^GZMB^−^ CD4 and CD8 T cells is associated with unfavorable prognosis in MDA5^+^DM. More in-depth studies are needed to explore the roles of cytotoxic T cells in MDA5^+^DM.
